# Full-Mouth Implant Rehabilitation of a Failing Dentition Using the All-on-6 Concept: A Clinical Case Report

**DOI:** 10.7759/cureus.116806

**Published:** 2026-09-23

**Authors:** Supriya Singh, Shubham K Srivastava, Akshim Rana, Shitij Srivastava, Shilpi Asthana

**Affiliations:** 1 Department of Prosthodontics, Sardar Patel Post Graduate Institute of Dental and Medical Sciences, Lucknow, IND; 2 Department of Periodontology, Swami Vivekanand Subharti University, Meerut, IND

**Keywords:** all-on-6, dental implants, failing dentition, full-mouth rehabilitation, implant-supported prosthesis

## Abstract

Full-mouth rehabilitation of patients with a failing dentition presents a complex clinical challenge, particularly when multiple teeth have compromised periodontal and prosthetic prognosis. This case report describes the prosthodontic rehabilitation of a 60-year-old male patient who reported to the department with a severely compromised dentition and a need for comprehensive oral rehabilitation. Following clinical and radiographic evaluation, treatment planning was undertaken to restore function, esthetics, and oral health using an implant-supported full-mouth rehabilitation based on the All-on-6 concept. The treatment involved comprehensive assessment, strategic implant placement, and prosthetic rehabilitation to achieve a stable and functional occlusion. The prosthetic phase was designed to provide improved masticatory efficiency, esthetics, and patient comfort while facilitating long-term maintenance. The case demonstrates the application of the All-on-6 concept as a treatment option for full-mouth rehabilitation in a patient with a failing dentition. Careful diagnosis, prosthetically driven treatment planning, appropriate implant positioning, and coordinated surgical and prosthetic management are essential for achieving predictable functional and esthetic outcomes.

## Introduction

A failing dentition represents a complex clinical condition characterized by loss of tooth structure, compromised periodontal support, impaired masticatory function, and progressive deterioration of oral health. In patients with multiple teeth exhibiting a poor or questionable prognosis, comprehensive rehabilitation requires careful evaluation of the remaining dentition, supporting structures, functional requirements, esthetic expectations, and long-term maintenance considerations. When preserving the natural dentition is no longer feasible, implant-supported rehabilitation can provide a fixed treatment option to restore oral function and patient satisfaction [1,2].

Implant-supported full-mouth rehabilitation has evolved substantially with advances in implant design, surgical planning, and prosthetic concepts. Full-arch fixed dental prostheses supported by multiple implants can provide stable prosthetic support and facilitate comprehensive functional and esthetic rehabilitation in appropriately selected patients [1,2]. Treatment planning should be prosthetically driven and should consider the available bone, implant distribution, interarch relationships, occlusion, restorative space, esthetic requirements, and the patient's ability to maintain adequate oral hygiene [1,3].

The All-on-6 concept uses six strategically distributed implants to support a full-arch fixed prosthesis. Six implants provide broad distribution across the arch and have been investigated as an approach for full-arch rehabilitation of edentulous jaws. Clinical evidence has demonstrated favorable long-term implant survival and prosthetic outcomes with six-implant-supported full-arch rehabilitations [4-5]. In a five-year randomized controlled trial comparing four- and six-implant-supported immediately loaded maxillary fixed prostheses, both treatment concepts demonstrated high clinical performance, although differences in implant survival and complications were not statistically significant [4]. Longer-term retrospective data have also demonstrated favorable implant survival for six-implant full-arch rehabilitation, supporting its use as a viable treatment option in appropriately selected patients [5].

Successful rehabilitation of a failing dentition, therefore, depends not only on implant placement but also on comprehensive diagnosis, prosthetically driven treatment planning, appropriate surgical-prosthetic coordination, and meticulous management of occlusion and esthetics [1,3]. This case report describes the comprehensive full-mouth implant rehabilitation of a 60-year-old male patient with a failing dentition using the All-on-6 concept, emphasizing the diagnostic assessment, treatment planning, implant-supported rehabilitation, and prosthodontic management involved in restoring oral function and esthetics.

## Case presentation

A 60-year-old male patient presented to the department with the chief complaint of difficulty in chewing food and dissatisfaction with the esthetics of his existing dentition. The patient reported no underlying systemic medical condition. Clinical examination revealed a severely compromised partially dentulous state involving both the maxillary and mandibular arches. The maxillary residual dentition comprised teeth 15, 14, 13, 12, 23, 25, 26, and 27, whereas the mandibular residual dentition comprised teeth 44, 43, 42, 31, 32, 33, 34, and 37. Teeth 32 and 34 exhibited Grade III mobility, while the remaining mandibular teeth were present as retained root stumps. The remaining natural teeth demonstrated poor periodontal status and an unfavorable prognosis for long-term tooth-supported rehabilitation.

Preoperative clinical and panoramic radiographic evaluation demonstrated extensive loss of the remaining dentition, multiple retained root stumps, and marked loss of alveolar bone support around the residual teeth (Figure 1A). Clinical and radiographic evaluation assessed overall oral and dental status and facilitated treatment planning. Cone-beam computed tomography (CBCT) imaging was not considered in this case, as the available clinical and panoramic radiographic findings provided sufficient information regarding the relevant anatomical structures and implant sites. Based on a comprehensive assessment of the available diagnostic records, we planned the surgical procedure accordingly. After evaluating the patient's functional and esthetic requirements and discussing the available treatment options, we planned full-mouth implant-supported rehabilitation using the All-on-6 concept for both arches.

**Figure 1:**
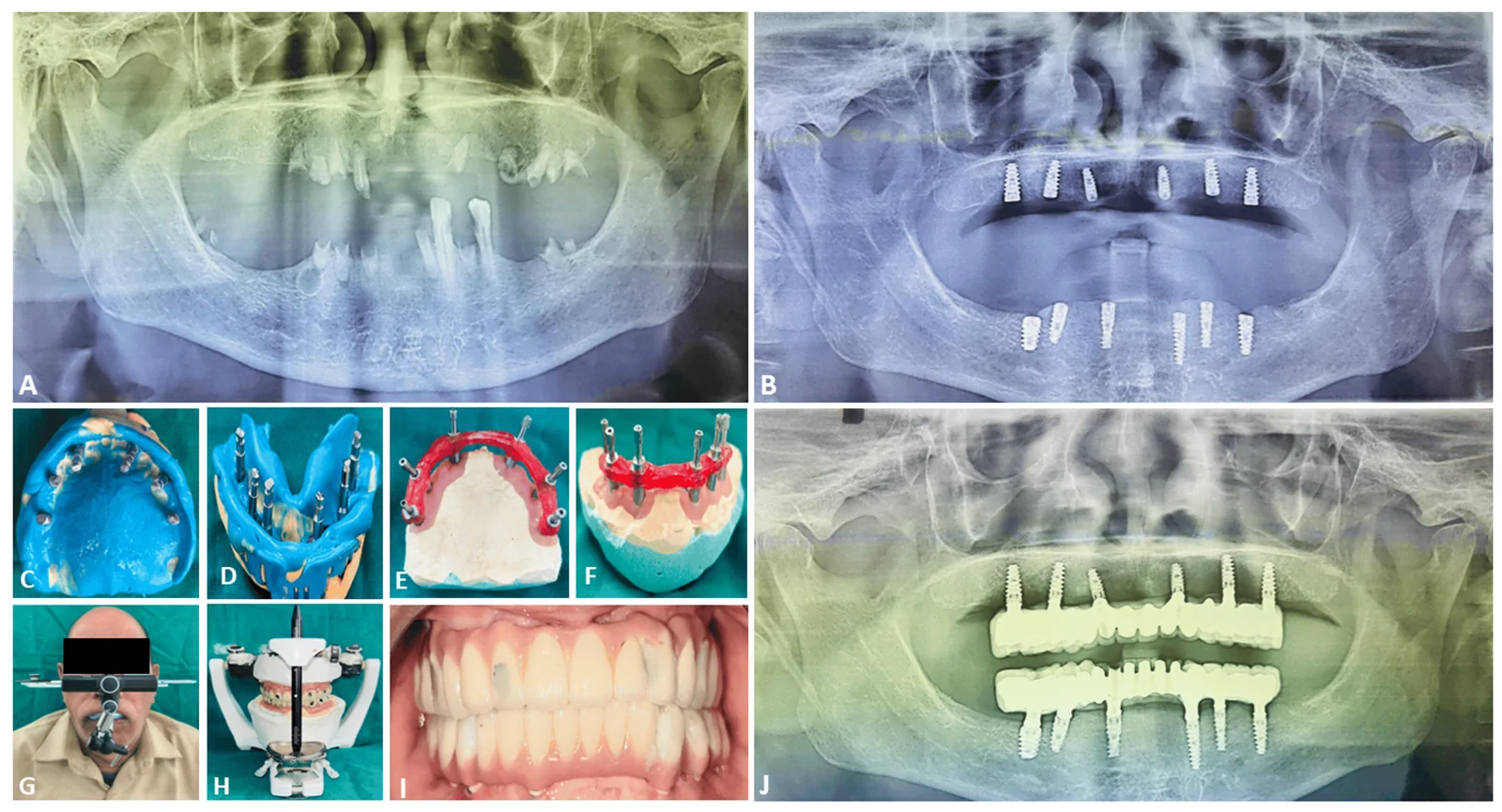
Clinical and prosthodontic workflow for full-mouth implant rehabilitation using the All-on-6 concept A: Preoperative panoramic radiograph demonstrating the failing dentition; B: postoperative panoramic radiograph demonstrating the placement of implants in the maxillary and mandibular arches; C: maxillary open-tray implant impression; D: mandibular open-tray implant impression; E: fabrication of the maxillary verification jig; F: fabrication of the mandibular verification jig; G: face-bow transfer; H: mounting on a semi-adjustable articulator and prosthetic fabrication; I: intraoral view following insertion of the definitive prostheses; J: post-prosthetic panoramic radiograph demonstrating the implant-supported full-mouth rehabilitation.

Before implant placement, the available prosthetic space was clinically evaluated in both arches by assessing the existing interarch relationship, vertical restorative space, occlusal relationship, and the requirements of the planned full-arch prosthesis. All remaining natural teeth and retained root stumps were subsequently extracted because of their poor prognosis, and no alveolar bone reduction was performed in either arch, as the available bone and prosthetic space were considered adequate for the planned rehabilitation. Implant-supported rehabilitation was then planned using six implants in each arch. In the maxillary arch, implants were planned at sites 16, 14, 12, 22, 24, and 26, whereas in the mandibular arch, implants were planned at sites 46, 45, 42, 32, 34, and 36. Dental implants (Noris Medical Ltd., Nesher, Israel) were used, with implant dimensions of 4.2 × 10 mm, 3.75 × 10 mm, 3.3 × 10 mm, and 5 × 8 mm selected according to the individual implant site and available anatomy. Implant osteotomies were prepared according to the manufacturer's recommended surgical protocol, followed by placement of the implants at the planned sites. Primary implant stability was assessed intraoperatively by recording the insertion torque at the time of implant placement. The insertion torque values ranged from 35 to 50 Ncm, indicating satisfactory primary mechanical stability of the implants. Despite the favorable primary stability achieved at implant placement, an immediate-loading protocol was not adopted. A delayed-loading protocol was selected to permit an extended period of undisturbed osseointegration and biological maturation of the peri-implant tissues. The implants were therefore maintained in an unloaded state for six months before initiation of the definitive prosthetic rehabilitation. Both immediate (12, 14, 26, 32, 34, 42) and delayed implant (16, 22, 24, 36, 45, 46) placement protocols were utilized according to the clinical site. No bone augmentation procedure was required or performed. Cover screws were placed, and all implants were submerged 0.5 mm to allow for undisturbed osseointegration. A postoperative panoramic radiograph confirmed the placement and distribution of the implants in both arches (Figure 1B).

A healing period of six months was allowed following implant placement to facilitate osseointegration. Following completion of the healing period, a second-stage surgical procedure was performed. The implants were surgically exposed, the cover screws were removed, and healing abutments were placed. The healing abutments were maintained for approximately 15 days to facilitate peri-implant soft-tissue maturation and establishment of an adequate peri-implant gingival collar before initiation of the definitive prosthetic phase. Following completion of the soft-tissue healing phase, the healing abutments were removed, and the peri-implant tissues were evaluated clinically for adequate maturation and formation of a stable peri-implant gingival collar. After confirmation of satisfactory soft-tissue healing, prosthodontic procedures were initiated.

Following completion of the soft-tissue healing phase, the healing abutments were removed, and engaging hex-type open-tray impression copings (Noris Medical Ltd., Nesher, Israel) were connected to each implant fixture and hand-tightened. Complete seating of the impression copings was verified clinically and, where indicated, radiographically using periapical radiographs. For the full-arch definitive impression, the impression copings were rigidly splinted using autopolymerizing pattern resin (GC Pattern Resin, GC Corporation, Japan). The splint was sectioned with a thin disc, and the segments were subsequently reconnected intraorally using small increments of pattern resin to minimize the potential effects of polymerization shrinkage and maintain the recorded three-dimensional interimplant relationship. A rigid custom tray was then used for the definitive open-tray impression, with appropriately positioned access openings to allow complete passage and subsequent removal of the guide screws. Polyvinyl siloxane (PVS) elastomeric impression material (Zhermack Elite HD+, Zhermack S.p.A., Italy) was used, with light-body PVS syringed around the impression copings and peri-implant soft-tissue interfaces while the tray was loaded with the appropriate PVS impression material. The tray was completely seated and maintained in a stable position until polymerization of the impression material. Following setting, the individual guide screws were completely loosened through the tray access openings before the impression was carefully removed to minimize distortion or displacement of the impression copings. The healing abutments were subsequently replaced. Implant analogs were connected to the corresponding impression copings within the definitive impressions, and definitive working casts were fabricated (Figure 1C-1D). Implant-specific verification jigs were subsequently fabricated on the definitive working casts using pattern resin (Figure 1E-1F). The verification jigs were sectioned and intraorally reconnected with pattern resin to verify the accuracy of transfer of the three-dimensional implant relationship to the definitive working casts before proceeding with framework fabrication and the definitive prosthetic procedures.

A facebow transfer was performed to establish the spatial relationship of the maxillary arch to the articulator (Figure 1G). The facebow record was transferred to a semi-adjustable articulator (Bio-Art A7 Plus; Bio-Art, Brazil), and the maxillary cast was mounted accordingly. The mandibular cast was subsequently mounted using the verified intermaxillary relationship at the established occlusal vertical dimension. The centric relation record was repeated to confirm reproducibility before definitive mounting.

Protrusive and right- and left-lateral eccentric interocclusal records were subsequently obtained intraorally and used for programming the semi-adjustable articulator. The protrusive record was used to establish the horizontal condylar inclination, whereas the lateral records were used to determine the lateral condylar guidance parameters. The articulator was programmed to reproduce the recorded mandibular excursions. The occlusal vertical dimension was established at 66 mm, with an interocclusal rest space of approximately 4 mm. The recorded eccentric relationships resulted in a horizontal condylar inclination of 30° bilaterally and a Bennett angle of 16° bilaterally.

Following definitive mounting and articulator programming, the prosthetic frameworks for the implant-supported porcelain-fused-to-metal (PFM) restorations were fabricated according to the planned implant positions, restorative space, and prosthetic contours. A screw-retained implant-supported PFM prosthetic design was selected based on the planned fixed full-arch rehabilitation, implant distribution, available restorative space, and anticipated long-term maintenance requirements. Screw retention provided direct attachment of the definitive prostheses to the implant-supported components and facilitated retrievability for future clinical evaluation, maintenance, repair, or management of prosthetic and implant-related complications. It also eliminated the need for luting cement and thereby avoided potential concerns associated with residual subgingival cement around the peri-implant tissues. A cement-retained design was not selected because the planned implant positions permitted screw-retained restoration without compromising the intended esthetic, functional, or occlusal objectives. A hybrid denture prosthesis was not selected because the treatment plan involved a fixed implant-supported PFM rehabilitation rather than a denture-based implant-supported prosthesis. The definitive prosthetic design was therefore selected to provide a fixed, retrievable restoration with appropriate emergence profiles, cleansable contours, adequate restorative space, and favorable force distribution.

An intraoral framework try-in was performed to evaluate the accuracy of the framework fabrication and its passive adaptation to the implant-supported components. Complete seating and stability of each framework were verified clinically, with particular attention to the implant-framework interfaces, absence of rocking or binding, and adaptation without clinically detectable discrepancies. The interarch relationship, restorative space, occlusal clearance, and relationship between the frameworks and opposing arch were also evaluated before proceeding with the ceramic veneering phase.

Following framework verification, the prosthetic teeth and occlusal morphology were established in accordance with the recorded maxillomandibular relationship and programmed articulator settings (Figure 1H). An implant-protective mutually protected occlusal scheme was developed, characterized by stable and evenly distributed centric contacts, appropriate anterior guidance, and controlled posterior contacts during eccentric mandibular movements. The occlusal morphology was designed with controlled cusp inclinations and appropriately reduced occlusal table dimensions to facilitate predominantly axial transmission of occlusal forces and minimize excessive lateral and non-axial loading of the implant-supported restorations. Posterior occlusal contacts were established to provide functional stability while avoiding premature or excessive eccentric contacts. The recorded protrusive and lateral eccentric relationships were reproduced on the programmed articulator to facilitate development of harmonious anterior guidance and posterior occlusal morphology.

Following completion and intraoral insertion of the definitive PFM restorations, complete seating, marginal adaptation, and stability of the prostheses were clinically verified. The occlusion was subsequently evaluated in maximum intercuspation and during protrusive and right- and left-lateral mandibular excursions. Occlusal contacts were assessed using articulating paper and further verified with shimstock foil. Particular attention was given to the identification of premature contacts, deflective occlusal interferences, excessive occlusal contacts, and unfavorable non-axial loading of the implant-supported restorations. Selective occlusal adjustment was performed where indicated to achieve stable and evenly distributed centric contacts and smooth, interference-free eccentric movements while preserving the planned occlusal scheme and functional morphology of the definitive restorations.

The prosthetic contours, tooth arrangement, midline, occlusal plane, esthetic integration, phonetics, lip support, and patient comfort were subsequently evaluated. After satisfactory clinical verification of the fit, occlusion, esthetics, and functional parameters, the prostheses were definitively secured, and the screw-access channels were sealed with an appropriate restorative material (Figure 1I).

A postoperative panoramic radiograph was obtained following definitive prosthesis insertion to assess the overall implant-supported rehabilitation and to verify the relationship of the implants with the corresponding prosthetic components (Figure 1J).

Following definitive prosthesis insertion, the patient was enrolled in a structured follow-up and maintenance program, with clinical evaluations performed at 1, 3, 6, and 12 months. At each follow-up visit, all 12 implants (six in the maxilla and six in the mandible) remained clinically stable and functional, with no implant loss or identified biological or mechanical complications. The implant-supported prostheses were evaluated for stability, occlusal harmony, integrity of the prosthetic components, and the presence of any mechanical or biological complications. The peri-implant soft tissues were clinically assessed, and oral hygiene instructions were reinforced as required. Radiographic evaluation was performed to monitor the peri-implant bone levels and the implant-prosthesis complex.

Throughout the 12-month follow-up period, the patient demonstrated a favorable clinical course. At the 12-month evaluation, all 12 implants and the definitive prostheses remained clinically stable, with no identified mechanical or biological complications. Radiographic assessment revealed no evident peri-implant bone loss. The patient reported satisfactory masticatory function and esthetics and expressed satisfaction with the definitive rehabilitation. The initial complaints of difficulty in chewing and esthetic dissatisfaction were resolved following treatment. Overall, the patient demonstrated satisfactory functional, esthetic, and radiographic outcomes during the 12-month observation period.

## Discussion

Management of a severely compromised dentition requires individualized assessment of tooth prognosis, periodontal support, residual alveolar anatomy, restorative space, interarch relationship, functional demands, esthetic expectations, and the patient's ability to maintain the definitive rehabilitation. In the present case, the residual dentition was characterized by poor periodontal support, grade III mobility of selected teeth, retained root stumps, and an unfavorable long-term prognosis. Consequently, preservation of the remaining dentition was considered unsuitable for comprehensive tooth-supported rehabilitation, and extraction followed by implant-supported full-arch rehabilitation was selected after discussion of the available treatment alternatives.

The All-on-6 concept was selected to provide distributed implant support for a fixed full-arch prosthesis in each arch. The number and distribution of implants should, however, be determined according to the anatomical and prosthetic requirements of the individual patient rather than by applying a predetermined implant number universally. Factors including available bone, implant distribution, anteroposterior spread, restorative space, prosthetic design, occlusal requirements, and maintenance considerations should be incorporated into treatment planning [1,2]. Therefore, in the present case, the use of six implants in each arch was a treatment decision based on the clinical and prosthetic requirements of the patient.

No bone augmentation was required in the present case, and implant positioning was adapted to the available anatomy. This observation should be interpreted cautiously and should not be generalized to patients with inadequate bone volume. Three-dimensional imaging such as CBCT can provide additional information regarding buccolingual bone dimensions and the relationship of proposed implant sites to relevant anatomical structures. CBCT was not performed in this case, and this represents a limitation of the preoperative diagnostic documentation. Accordingly, the favorable outcome observed in this patient should not be interpreted as evidence that panoramic radiography is sufficient for all extensive full-arch implant rehabilitations.

Both immediate and delayed implant placement were used according to the individual clinical sites. The choice between immediate and delayed placement should be based on local anatomical and clinical conditions, including the extraction-site morphology, residual bone availability, infection status, primary stability requirements, and prosthetic considerations. Evidence indicates that predictable implant outcomes can be achieved with both approaches when appropriate case selection and treatment protocols are followed [6]. In the present case, the timing of implant placement was therefore determined on a site-specific basis rather than by adherence to a single placement protocol.

Primary implant stability is an important factor in determining the feasibility of immediate loading. In the present case, insertion torque values ranged from 35 to 50 Ncm. Although such values may permit immediate loading in appropriately selected full-arch cases, immediate loading was not pursued. A delayed-loading protocol was intentionally selected to allow undisturbed osseointegration, with the implants submerged for six months before prosthetic rehabilitation. Thus, the decision to delay loading was a case-specific clinical choice and should not be interpreted as an indication that the recorded insertion torque values were inadequate for immediate loading. Evidence indicates that immediate, early, and conventional loading can all achieve favorable outcomes in appropriately selected edentulous patients, although outcomes are influenced by implant stability, implant distribution, bone quality, splinting, prosthetic design, and patient-related factors [7,8].

The accuracy of implant-position transfer is particularly important in full-arch rehabilitation because discrepancies between the intraoral implant positions and those reproduced on the definitive working cast may adversely affect framework adaptation and the implant-prosthesis interface. In the present workflow, definitive implant-level impressions were obtained using an open-tray technique with custom trays and engaging hex-type impression copings. Implant analogs were subsequently connected to the corresponding impression copings within the definitive impressions, and definitive working casts were fabricated. Implant-specific verification jigs were then fabricated on the definitive working casts using pattern resin. The jigs were sectioned, and the segments were reconnected intraorally with pattern resin to reproduce the three-dimensional intraoral implant relationship and minimize the potential effects of polymerization shrinkage. The verified jig was subsequently evaluated for complete seating and stability before proceeding with definitive prosthetic procedures. The accuracy of implant impressions may be influenced by several factors, including impression material, tray rigidity and design, splinting technique, implant angulation, impression coping characteristics, and the clinical technique employed.

The prosthodontic phase incorporated face-bow transfer, intermaxillary records, and protrusive and lateral eccentric records for programming of the semi-adjustable articulator. These records facilitated transfer of the maxillomandibular relationship and development of the planned occlusal scheme. Occlusal verification was subsequently performed during the bisque trial, including assessment of maximum intercuspation and eccentric movements, with adjustments made where required. Such systematic recording and verification are particularly important in full-arch implant rehabilitation because errors in jaw relation, occlusal contacts, or prosthetic tooth arrangement may influence both function and patient comfort.

The definitive prostheses were fabricated as screw-retained PFM full-arch restorations. Screw retention was selected primarily because of its retrievability and the potential advantage of facilitating future maintenance and management of prosthetic complications. Avoidance of cement-related complications around multiple implants was an additional consideration. Nevertheless, screw-retained restorations are associated with their own technical complications, including screw loosening, screw fracture, and veneering or ceramic complications [9]. Therefore, the choice between screw-retained, cement-retained, and hybrid prosthetic designs should be individualized according to implant position, restorative space, prosthetic design, esthetic requirements, retrievability, and maintenance considerations.

Esthetic assessment was performed during the bisque trial before definitive processing. Tooth arrangement, midline, incisal display, tooth proportions, shade, phonetics, facial support, soft-tissue contours, and occlusal relationships were evaluated, and patient approval was obtained before final processing. Although the definitive prosthesis achieved satisfactory function and patient-reported esthetics in this case, the esthetic outcome should be interpreted within the limitations of a single clinical report. Clinical photographs may also be influenced by factors such as photographic angulation, lighting, lip position, and facial expression and therefore should not be considered an objective measure of esthetic success in isolation.

The patient was followed at one, three, six, and 12 months after definitive prosthesis insertion. At the 12-month evaluation, the prostheses remained clinically stable, with no identified mechanical or biological complications and no radiographically evident peri-implant bone loss. The patient reported improvement in masticatory function and esthetic satisfaction. These findings represent the short-term outcome observed in this individual patient. They cannot be extrapolated to long-term implant or prosthetic survival because biological and technical complications may develop with extended function [10]. Continued maintenance and periodic clinical and radiographic assessment therefore remain essential.

The principal strength of this report is the description of a comprehensive bilateral full-arch rehabilitation involving coordinated surgical and prosthodontic procedures, including site-specific implant placement, delayed loading, definitive open-tray impressions, implant-position verification, articulator-based jaw-relation procedures, eccentric records, and screw-retained PFM prostheses. However, several limitations must be acknowledged. This is a single case with a 12-month follow-up, and therefore it cannot establish the predictability or superiority of the All-on-6 concept compared with alternative full-arch rehabilitation protocols. Furthermore, CBCT was not performed, limiting three-dimensional documentation of the preoperative implant-site anatomy. The esthetic and functional outcomes are also patient-specific and should not be generalized.

Overall, this case illustrates the clinical application of a prosthetically oriented All-on-6 treatment workflow for a patient with a severely compromised dentition. The satisfactory 12-month outcome suggests that, in this individual case, careful diagnosis, individualized implant positioning, controlled loading, verification of implant relationships, accurate definitive impressions, comprehensive jaw-relation records, and systematic prosthetic evaluation contributed to the successful delivery of the rehabilitation. Longer-term follow-up and evidence from comparative clinical studies are required to determine the durability and relative effectiveness of this treatment approach.

## Conclusions

In the present case, full-mouth implant rehabilitation using the All-on-6 concept resulted in satisfactory functional and esthetic outcomes in a patient with a severely compromised, failing dentition. Following extraction of the remaining teeth and retained root stumps, six implants were placed in each arch using a combination of immediate and delayed implant placement according to the clinical site and without bone augmentation. Although insertion torque values ranged from 35 to 50 Ncm, a delayed-loading protocol with submerged healing was selected to allow undisturbed osseointegration, followed by second-stage surgery and prosthetic rehabilitation with screw-retained porcelain-fused-to-metal full-arch implant-supported prostheses. At the 12-month follow-up, all 12 implants and the definitive prostheses remained clinically stable, with no identified mechanical or biological complications, and the panoramic radiograph demonstrated no radiographically evident peri-implant bone loss. The patient reported resolution of the initial functional and esthetic concerns, with satisfactory masticatory function and esthetics. This case illustrates the clinical application of an appropriately planned All-on-6 approach incorporating individualized implant positioning, verification of implant relationships, controlled prosthetic fabrication, comprehensive jaw-relation records, and structured follow-up. However, as this is a single case with a 12-month follow-up, these findings represent a satisfactory short-term, patient-specific outcome and should not be interpreted as evidence of long-term treatment success, predictability, or superiority of the All-on-6 concept over alternative full-arch implant rehabilitation protocols. Longer-term follow-up and comparative clinical studies are required to evaluate the durability and relative effectiveness of this treatment approach.
